# A spike-targeting bispecific T cell engager strategy provides dual layer protection against SARS-CoV-2 infection in vivo

**DOI:** 10.1038/s42003-023-04955-3

**Published:** 2023-06-01

**Authors:** Fanlin Li, Wei Xu, Xiaoqing Zhang, Wanting Wang, Shan Su, Ping Han, Haiyong Wang, Yanqin Xu, Min Li, Lilv Fan, Huihui Zhang, Qiang Dai, Hao Lin, Xinyue Qi, Jie Liang, Xin Wang, Shibo Jiang, Youhua Xie, Lu Lu, Xuanming Yang

**Affiliations:** 1 Sheng Yushou Center of Cell Biology and Immunology, School of Life Sciences and Biotechnology, Shanghai Jiao Tong University, Shanghai, 200240 China; 2 Joint International Research Laboratory of Metabolic & Developmental Sciences, Shanghai Jiao Tong University, Shanghai, 200240 China; 3 Key Laboratory of Medical Molecular Virology (MOE/NHC/CAMS), Shanghai Institute of Infectious Disease and Biosecurity, School of Basic Medical Sciences and Biosafety Level 3 Laboratory, Fudan University, Shanghai, 200032 China; 4 Department of Physiology, Naval Medical University, Shanghai, 200433 China; 5 Shanghai Longyao Biotechnology Limited, Shanghai, 201203 China

**Keywords:** SARS-CoV-2, Immunotherapy

## Abstract

Neutralizing antibodies exert a potent inhibitory effect on viral entry; however, they are less effective in therapeutic models than in prophylactic models, presumably because of their limited efficacy in eliminating virus-producing cells via Fc-mediated cytotoxicity. Herein, we present a SARS-CoV-2 spike-targeting bispecific T-cell engager (S-BiTE) strategy for controlling SARS-CoV-2 infection. This approach blocks the entry of free virus into permissive cells by competing with membrane receptors and eliminates virus-infected cells via powerful T cell-mediated cytotoxicity. S-BiTE is effective against both the original and Delta variant of SARS-CoV2 with similar efficacy, suggesting its potential application against immune-escaping variants. In addition, in humanized mouse model with live SARS-COV-2 infection, S-BiTE treated mice showed significantly less viral load than neutralization only treated group. The S-BiTE strategy may have broad applications in combating other coronavirus infections.

## Abstract (web summary)

A SARS-CoV-2 spike-targeting bispecific T-cell engager (S-BiTE) strategy is presented that effectively reduces SARS-CoV-2 viral load in a humanized mouse model.

## Introduction

The emergence of the novel human coronavirus SARS-CoV-2 has caused a worldwide pandemic of coronavirus disease 2019 (COVID-19), hampering health and economic systems globally^1–3^. Different vaccination formats have been demonstrated to confer protection against the original or mutated SARS-CoV-2 strains at different efficacies. The mRNA-based, adenoviral vector-based, and inactivated virus-based vaccines are reported to be 95%, 66.9%, and 65.9% effective, respectively, in preventing COVID-19^4–6^. Despite the availability of these working vaccines, the emergence of immune-escaping variants significantly slowed the controlling of the pandemic^7,8^.

Various small molecules targeting SARS-CoV-2 entry or replication in cells are under evaluation^9^ and some of them have been authorized for clinic usage^10^. Among them, Paxlovid has shown clinical benefit of reducing the risk of progression to severe COVID-19 or death^11–13^. Neutralizing antibodies have been used clinically against respiratory syncytial virus and Ebolavirus disease^14,15^, and also quickly been developed against SARS-CoV-2 infection to exert potent neutralizing activity in preclinical models and clinic^15–19^. Angiotensin-converting enzyme 2 (ACE2) is a key entry receptor for SARS-CoV-2^1^. Several studies have reported the antiviral effects of soluble ACE2-based therapeutics, functioning as a competitive inhibitor of membranous ACE2^20–23^. To further enhance the efficacy, bispecific antibodies targeting two epitopes^24–27^ and bispecific fusion proteins with antibody arm and soluble ACE2 arm^28,29^ have been developed to enhance neutralization to prevent escaping mutation of SARS-CoV-2. However, owing to the high mutation rate of SARS-CoV-2, these therapeutics will face challenges of treatment-escape or resistance eventually. Furthermore, spike mutations have been reported to be associated with the reduced neutralization ability of monoclonal antibodies and serum antibodies in vaccinated and convalescent individuals^30–38^. Therefore, to achieve the optimal therapeutic potential, a novel approach, in addition to the utilization of neutralizing antibodies and small molecular inhibitors, needs to be developed. To address this need, in this paper, we present a SARS-CoV-2 spike-targeting bispecific T-cell engager (S-BiTE) strategy for controlling SARS-CoV-2 infection.

## Results

### Generation and characterization of S-BiTE

**Fig. 1:**
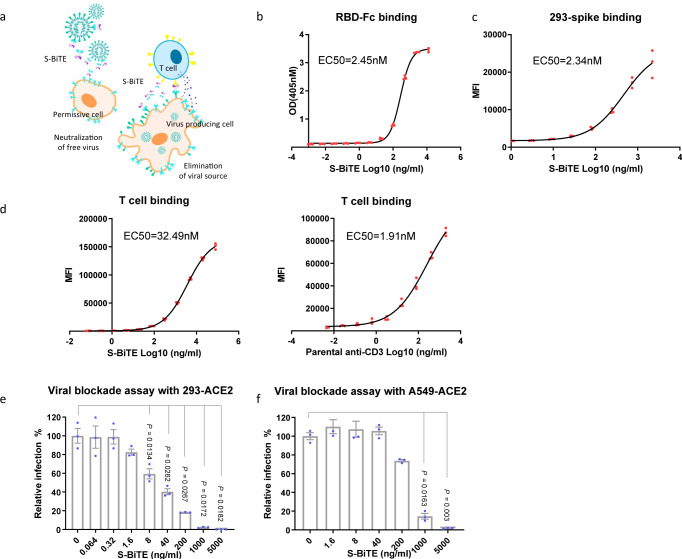
Generation and characterization of the S-BiTE fusion protein. **a** A schematic diagram of the potential anti-SARS-CoV-2 mechanism of the spike-targeting bispecific T cell engager (S-BiTE) used in this study. **b** ELISA-binding curves of S-BiTE to immobilized RBD of the SARS-CoV-2 spike. **c** MFI of the binding of S-BiTE to 293-spike cells as determined by flow cytometry. **d** Median fluorescence intensity (MFI) of the binding of S-BiTE or parental anti-CD3 to primary human T cells. **e**, **f** Lentiviruses pseudotyped with the SARS-CoV-2 spike were incubated with 293-ACE2 cells (**e**) or A549-ACE2 cells (**f**) in the presence of indicated concentration of S-BiTE. Fluorescent IRFP-positive cells were measured by flow cytometry. Relative infection was calculated as ratio of the IRFP readout in the presence of S-BiTE to the IRFP readout in the absence of S-BiTE. One-way ANOVA with Dunnett’s multiple comparison and correction was performed and significance was shown. All data shown as mean ± SEM. Representative results from one of three repeated experiments are shown (*n* = 3/group) (**b**–**f**).

T cells are considered to be the most effective immune cells in eliminating virus-infected cells or cancer cells^39,40^. BiTE, a potent T cell-activation strategy, has been widely used for treating various types of cancer^41^, but little is known about its effect on SARS-CoV-2 infection. Thus, we designed a fusion protein, S-BiTE, consisting of the ACE2 extracellular domain (amino acid 1–740) to block viral entry and the anti-CD3ε single-chain variable fragment (scFv) to activate T cells and eliminate viral-producing cells (Fig. 1a and supplementary Fig. 1a).

SARS-CoV-2 enters permissive cells through ACE2 in a SARS-CoV-2 spike-dependent manner^1,42^. Owing to the specific and strong interaction between ACE2 and SARS-CoV-2 spike^43^, we used the extracellular domain of ACE2 as a specific ligand to identify spike-expressing cells, which mimic SARS-CoV-2-infected cells in vivo. This monovalent ACE2 extracellular domain has a relatively high affinity for the receptor binding domain (RBD) of spike-Fc fusion protein and spike-expressing 293-spike cells (Fig. 1b, c). The other portion of the fusion protein is the monovalent anti-CD3ε scFv portion that showed a significantly reduced affinity for CD3ε compared with the parental bivalent anti-CD3 antibody (Fig. 1d and Supplementary Fig. 1b), which disfavors the binding and activation of T cells in the absence of the SARS-CoV-2 spike^44,45^.

The ACE2 portion of S-BiTE could function as a competitive receptor and entry blocker of SARS-CoV-2. To test this hypothesis, we performed a pseudotyped SARS-CoV-2 blockade assay in vitro and found that S-BiTE significantly blocked pseudotyped SARS-CoV-2 infection in permissive 293-ACE2 and A549-ACE2 cells (Fig. 1e–f).

### S-BiTE induced target-dependent T cell activation and cytotoxicity in the presence of the SARS-CoV-2 spike

**Fig. 2:**
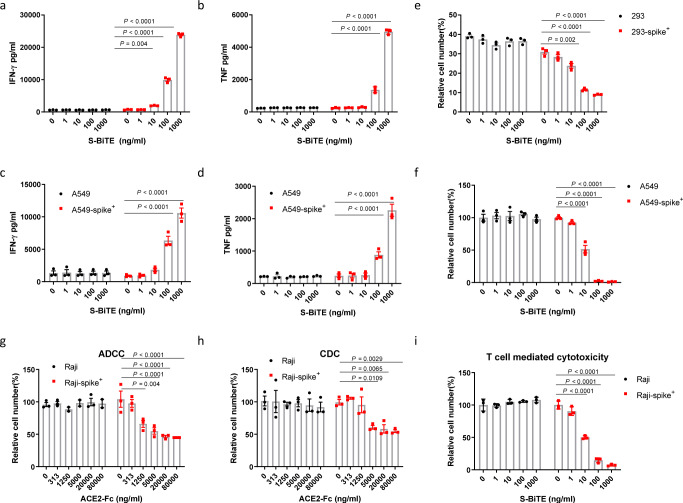
S-BiTE induced target-dependent T cell activation and cytotoxicity in the presence of spike. 293, 293-spike, A549, A549-spike, Raji, or Raji-spike cells were co-cultured with human primary T cells in the presence of the indicated concentration of S-BiTE. **a**–**d** After 24 h, levels of IFN-γ and TNF in the cell supernatant were analyzed by the CBA assay. **e**, **f** After 48 h, cytotoxicity was determined by measuring CD45^-^ cells via flow cytometry. **h**, **i** Raji or Raji-spike cells were co-cultured with human primary NK cells and supplemented with human AB serum or human T cells in the presence of the indicated concentration of ACE2-Fc or S-BiTE. ADCC, CDC, or T cell-mediated cytotoxicity was analyzed by flow cytometry. Two-way ANOVA with Dunnett’s multiple comparison and correction was performed and significance was shown. All data shown as mean ± SEM. Representative results from one of three repeated experiments are shown (*n* = 3/group) (**a**–**i**).

The ability of S-BiTE to activate T cells was investigated in an in vitro T cell-activation assay with co-cultured cells engineered to express the SARS-CoV-2 spike. S-BiTE specifically activated T cells to release IFN-γ and TNF in the presence of 293-spike cells in a dose-dependent manner (Fig. 2a, b and Supplementary Fig. 2a, b). In the same experimental setting, there was no T cell activation in response to the spike-negative 293 cells even with the highest tested concentration of S-BiTE, suggesting the high specificity of the ACE2-spike interaction. The same specific and sensitive T cell activation was observed in response to spike-expressing lung epithelial A549 cells (Fig. 2c, d and Supplementary Fig. 2c, d). To further investigate whether the increased T cell activation could lead to death of the target cells, we performed a flow cytometry-based cell-killing assay. S-BiTE induced strong cytotoxicity towards the spike-expressing 293 and A549 cells, rather than towards the corresponding spike-negative control cells (Fig. 2e, f, Supplementary Fig. 2e, f, and Supplementary Fig. 3). To further confirm its specifity, we established one CD20 targeting BiTE and performed similar in vitro T cell activation assay, S-BiTE induce more T cell activation than control CD20-BiTE dependent on spike expression (Supplementary Fig. 5). These results demonstrate that S-BiTE can induce CD3-mediated activation of human T cells and the killing of SARS-CoV-2 spike-expressing cells.

Numerous studies have shown a strong inhibitory effect of neutralizing antibodies on viral entry into permissive cells^14,15,46,47^. However, only a few therapeutic neutralizing antibodies have been approved for clinical use^48^. The clinical efficacy of neutralizing antibodies may be limited by the emergence of viral mutations that enable escape from neutralization or the relatively weak ability of neutralizing antibodies to eliminate virus-infected cells. A recent publication showed that neutralization antibodies are less effective when administered 2 h after infection compared to their administration 24 h before infection^49^. Thus, we compared the cytotoxicity of S-BiTE and ACE2-human IgG1 Fc fusion proteins in vitro. Although IgG1 Fc is the most potent isotype in mediating antibody-dependent cell-mediated cytotoxicity (ADCC) and complement-dependent cytotoxicity (CDC)^50^, ACE2-Fc showed weak cytotoxic effects through ADCC and CDC in vitro (Fig. 2g, h). In the same setting, S-BiTE exhibited more than 2000 times higher killing effect on spike-expressing Raji cells (Fig. 2i).

### S-BiTE inhibited pseudotyped SARS-CoV-2 viral release

**Fig. 3:**
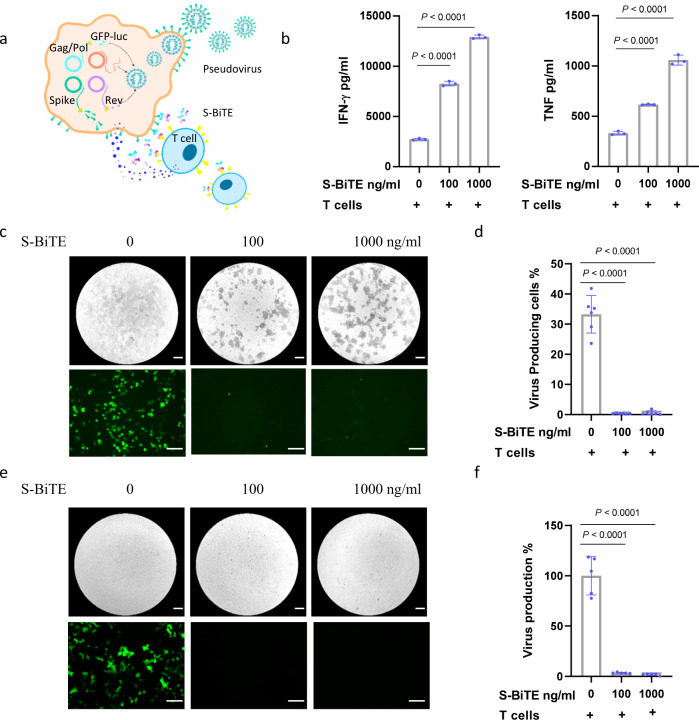
S-BiTE inhibited viral release in a pseudotyped SARS-CoV-2 production assay. **a** Schematic illustrating the co-culture experiments to monitor the effect of S-BiTE on virus-producing cells. **b**–**d** Lenti-X 293 T cells were transfected with pseudotyped SARS-CoV-2 plasmids. After 24 h, human primary T cells were added to the culture in the presence of the indicated concentration of S-BiTE. Forty-eight hours post transfection, levels of TNF and IFN-γ in supernatant were analyzed by the CBA assay (*n* = 3/group) (**b**). The remaining virus-releasing cells were imaged by fluorescent microscopy (**c**) and analyzed by flow cytometry (**d**) (*n* = 6/group). **e**, **f** The released virus in the supernatant was used to infect 293-ACE2 cells, and virus-infected GFP-expressing cells were analyzed by fluorescent microscopy (**e**) and flow cytometry (**f**) (*n* = 5/group). One-way ANOVA with Dunnett’s multiple comparison and correction was performed and significance was shown. Scale bar: 120 μm. All data shown as mean ± SEM. Representative results from one of three repeated experiments are shown (**b**–**f**).

The elimination of virus-infected cells to prevent the production of more viruses is critical for viral control, especially in the early stages of infection. Thus, we assessed the ability of S-BiTE to prevent virus release in our pseudotyped SARS-CoV-2 production system. To mimic virus-producing cells, 293 cells were transfected with four viral component-encoding plasmids (Fig. 3a). Upon co-culture with the engineered 293 cells, S-BiTE significantly triggered the activation of T cells, killing of viral-producing cells, and reduction of virus release (Fig. 3b–d). Importantly, under these conditions, the presence of free viruses in the culture medium did not affect S-BiTE-mediated T cell activation and cytotoxicity towards the spike-expressing cells. Consistently, the pseudotyped virus in the supernatant was significantly reduced after S-BiTE treatment (Fig. 3e, f). Collectively, these results indicate that S-BiTE is superior to the Fc-directed Ab therapeutic strategy in controlling viral infection at the cellular level.

### S-BiTE eliminated spike-expressing cells in vivo with good safety profile

**Fig. 4:**
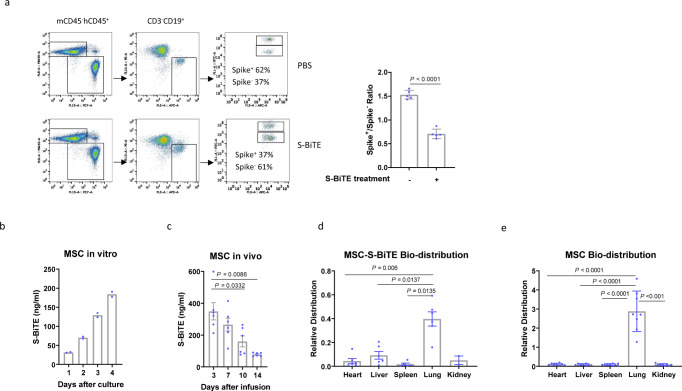
S-BiTE eliminated spike-expressing cells in vivo. **a** Approximately 2 × 10^6^ CFSE-labeled Raji and Raji-spike cells were mixed with 5 × 10^6^ T cells and intraperitoneally injected into NSG mice (*n* = 5/group). The mice were treated with PBS or S-BiTE, and cells in peritoneal cavity were collected and analyzed by flow cytometry 6 h after treatment. **b** The continuous S-BiTE production by stably engineered S-BiTE-MSCs was measured by ELISA after 1-month culture (*n* = 2/group). **c** S-BiTE-MSCs was injected to NSG mice (*n* = 6/group) and serum levels of S-BiTE were determined by ELISA at the indicated time points. **d**, **e** S-BiTE-MSCs (*n* = 6/group) (**d**) or MSCs (*n* = 9/group) (**e**) were injected into NSG mice, and bio-distribution in the indicated tissue was analyzed by q-PCR. Unpaired Student’s *T*-tests (**a**) and one-way ANOVA with Dunnett’s multiple comparison and correction (**b**–**e**) was performed and significance was shown. All data shown as mean ± SEM. **a**–**c** representative results from one of four repeated experiments are shown. **d**, **e** pooled results from three independent experiments are shown.

Given the potent in vitro cytotoxicity of S-BiTE towards spike-expressing cells, its cytotoxicity was also assessed in vivo. Similar to that of other reported BiTE-like molecules, the half-life of S-BiTE in mice is approximately 1.5 h (Supplementary Fig. 4). Despite its short half-life, a single treatment of S-BiTE was demonstrated to kill spike-positive target cells to a significant extent in the in vivo cell-killing assay, suggesting the high potential of S-BiTE to kill virus-infected cells in vivo (Fig. 4a and Supplementary Fig. 6). To test whether S-BiTE could induce unwanted T cell activation or T cell depletion, which is critical for its safety profile. hACE2 and hCD3e humanized mice were adminstrated with S-BiTE. Our data showed that there is no significant difference in immune cell subtypes, T cell activation marker and major tissue immune cell infiltration (Supplementary Fig. 7).

Furthermore, since mesenchymal stem cells (MSCs) have been widely used for treating various diseases and have been demonstrated to have good safety profiles in clinical settings^51^, in order to meet urgent clinical needs, we engineered MSCs to stably secrete S-BiTE (Fig. 4b). Serum expression of S-BiTE in mice lasted for more than 14 days after a single infusion (Fig. 4c). Importantly, when we checked the bio-distribution of MSCs, both engineered and unmodified MSCs were preferentially located in the lungs (Fig. 4d, e), suggesting their potential application in treating SARS-CoV-2-induced pneumonia. This MSC-based S-BiTE delivery approach provides a potential clinical ready option for the treatment of severe COVID-19 patients.

### S-BiTE eliminated live virus-infected cells

**Fig. 5:**
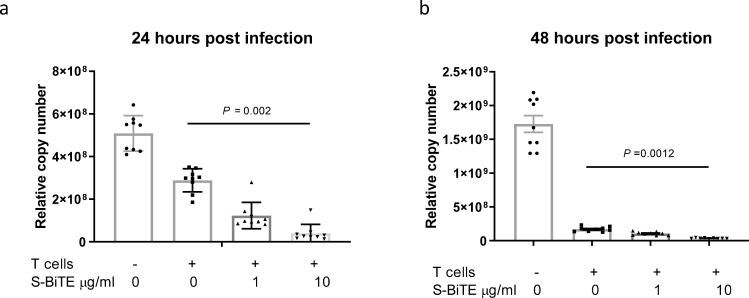
S-BiTE inhibited live SARS-CoV-2 replication in permissive cells. **a**, **b** A549-ACE2 cells were infected with live SARS-CoV-2 at an MOI of 0.05. After 2 h, free viruses were removed by washing with PBS, and human primary T cells were added to the culture in the presence of the indicated concentration of S-BiTE. At 24 h (**a**) and 48 h (**b**) post infection, SARS-CoV-2 replication in cells was analyzed by quantitative real-time PCR (*n* = 9/group). One-way ANOVA with and Dunnett’s multiple comparison and correction was performed and significance was shown. All data shown as mean ± SEM. Representative results from one of three experiments are shown (**a**, **b**).

Given the potential cytotoxicity of S-BiTE in eliminating spike-expressing cell lines, we further tested the efficacy of S-BiTE on live virus-infected cells. To establish infection, we infected A549-ACE2 cells with live SARS-CoV-2 for 2 h. Then, S-BiTE was added to the infected cells in the presence of T cells. Consistent with the results of the pseudovirus assay, S-BiTE significantly inhibited viral replication in permissive A549-ACE2 cells (Fig. 5a, b and Supplementary Fig. 5).

### S-BiTE is effective against the Delta variant of SARS-CoV-2

Owing to its high mutation rate, SARS-CoV-2 has evolved rapidly, leading to the emergence of many variants with immune-escape ability or enhanced proliferation and transmission capabilities worldwide. Among these variants, the Delta variant has been reported to increase the viral load in patients, contributing to the rapid global spread of this variant^52^. The efficacy of neutralization antibodies against the Delta variant has been reported to be 3 to 5 times lower than that against the original SARS-CoV-2 strain^30,38^. Thus, we investigated whether S-BiTE is effective against the Delta variant spike.

**Fig. 6:**
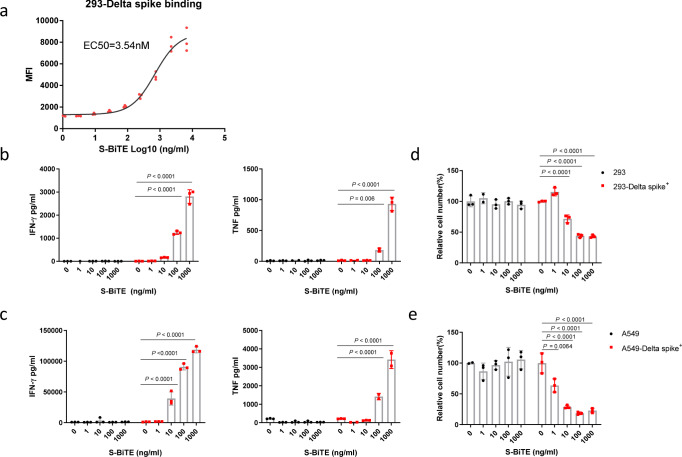
S-BiTE induced T cell activation and cytotoxicity against SARS-CoV-2 Delta-variant spike. **a** MFI of the binding of S-BiTE to 293-Delta spike cells was determined by flow cytometry. **b**–**e** 293, 293-Delta spike, A549, and A549-Delta spike cells were co-cultured with human primary T cells in the presence of the indicated concentration of S-BiTE. After 24 h, levels of IFN-γ and TNF in supernatant were analyzed by the CBA assay (**b**, **c**). After 48 h, cytotoxicity was determined by measuring CD45^-^ cells via flow cytometry (**d**, **e**). Representative results from one of three experiments are shown (*n* = 3/group) (**a**–**e**). Two-way ANOVA with Dunnett’s multiple comparison and correction was performed and significance was shown. All data shown as mean ± SEM.

We first tested the binding of S-BiTE to Delta-spike-expressing cells. S-BiTE bound to the 293-Delta-spike at an EC50 of 3.45 nM, which is similar to that observed for the WT spike (Fig. 6a). We then investigated whether S-BiTE could cause the lysis of Delta-spike-expressing cells in the presence of T cells. S-BiTE could specifically activate T cells to release IFN-γ and TNF in the presence of 293-Delta-spike cells or A549-Delta-spike cells in a dose-dependent manner (Fig. 6b, c). Consistent with the increased T cell activation, S-BiTE also induced strong cytotoxicity towards Delta-spike-expressing 293 and A549 cells, rather than towards the corresponding spike-negative control cells (Fig. 6d, e). These results demonstrate that S-BiTE can induce CD3-mediated activation of human T cells and kill cells expressing a mutated SARS-CoV-2 spike, which may be useful for the treatment of immune-escaping variants of SARS-CoV-2.

### S-BiTE shows better viral control ability in vivo than soluble ACE2 neutralizing agent

**Fig. 7:**
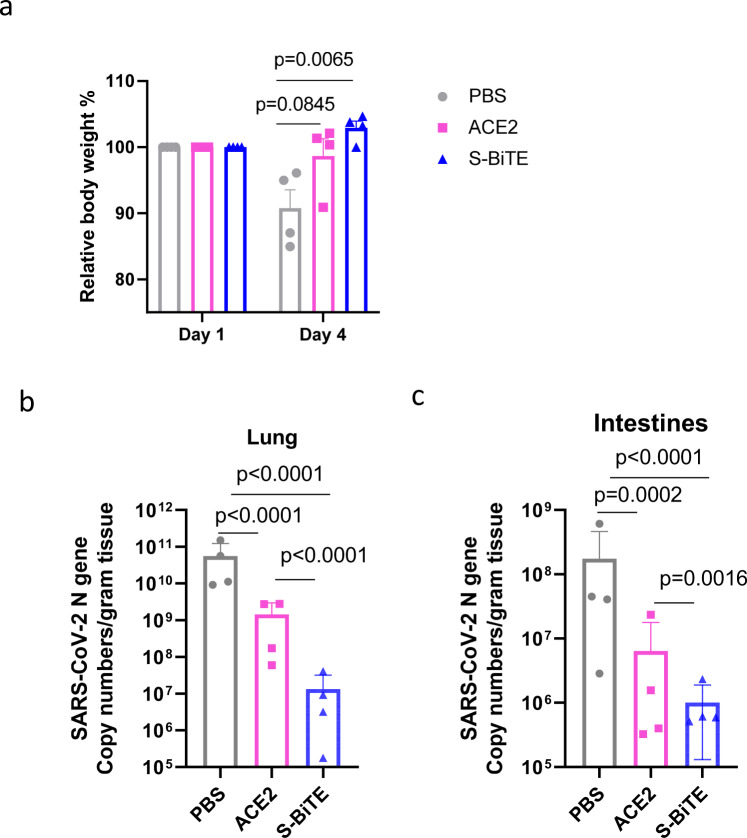
The protection efficiency of S-BiTE against SARS-CoV2 in hACE2-hCD3ε transgenic mice in vivo. **a**–**c** All hACE2-hCD3ε transgenic mice (*n* = 4/group) were challenged intranasally with SARS-CoV2 Delta-variant, and 25 μg of S-BiTE were intranasally administrated -1, 24, and 48 h post-infection. Equal mole of soluble ACE2 or equal volume of PBS was used as controls. **a** The body weight of mice was recorded and normalized at indicated time points. **b**, **c** The virus titer in lungs and intestines of three groups were determined at 3 dpi by qRT-PCR. Unpaired Student’s *T*-tests (**a**–**c**) was performed and significance was shown (*n* = 4/group). All data shown as mean ± SEM.

To explore the protection efficacy of S-BiTE against challenge with SARS-CoV2 Delta-variant virus in vivo, hACE2-hCD3ε transgenic mice were chosen as in vivo model. In hACE2-hCD3ε transgenic mice, hACE2 provides the entry receptor for SARS-CoV2 infection, and hCD3ε provides the T cell activating target by S-BiTE. To compare the protection efficacy of S-BiTE with neutralizing agent, a soluble ACE2-treated group was included as well. The body weight of ACE2 and S-BiTE group decreased significantly less compared with the PBS group (Fig. 7a). Since the lung and intestine are two major tissues-infected by SARS-CoV2 in this transgenic mouse, the number of viral RNA copies were measured and compared at 3 dpi. The RNA copies in the lung of both ACE2 group and S-BiTE group were significantly lower than the PBS group, reducing to about 10^−2^ and 10^−3^ of PBS group, respectively (Fig. 7b, c). Importantly, the RNA copies of S-BiTE group were reduced to about 10^−2^ of ACE2 group, suggesting the powerful protection by S-BiTE-mediated T cell activation, which is consistent with our in vitro observation. These results demonstrate that S-BiTE can induce both neutralization and CD3-mediated T cell activation in vivo, which provide dual layer protection against immune-escaping variants of SARS-CoV-2 and may be a potential treatment for COVID-19.

## Discussion

This study presents S-BiTE as an attractive therapeutic strategy for treating COVID-19 and other diseases caused by coronaviruses that use ACE2 as their receptor. S-BiTE could block viral entry by competing with the SARS-CoV-2 spike in binding to membrane ACE2. Furthermore, S-BiTE stimulated the powerful cytotoxic capabilities of T cells and increased sensitivity in eliminating virus-infected spike-expressing cells. This dual functional design can simultaneously prevent viral spread and reduce virus production.

Compared to the widely used neutralization-antibody strategy, the S-BiTE concept has two distinct advantages. The first advantage is the use of ACE2, the entry receptor for SARS-CoV-2, as a targeting moiety as theoretically, no SARS-CoV-2 mutant should be able to escape the ACE2-targeting S-BiTE treatment. However, owing to the different binding epitopes of neutralization antibodies, it is possible that the new mutants of SARS-CoV-2 are able to escape the predesigned neutralization-antibody treatment; antibodies from vaccinated and convalescent individuals have been reported to show reduced re-neutralization abilities against mutated SARS-CoV-2 variants^30–38^. Different approaches have been used to improve the ability of neutralization antibodies against escaping variants of SARS-CoV-2, such as antibody cocktails^53,54^ and bispecific antibodies^25,55^. Targeting multiple non-overlap epitopes including RBD can sufficiently improve affinity against spike protein and prevent escaping variants. One unique bispecific design, ACE-MAB (STI-4920)^28^, composes of a truncated extracellular ACE2 and an antibody against different epitope of SARS-CoV-2 spike. This design can simultaneously neutralize SARS-CoV-2 by competing membrane ACE2 and block CD147 binding to reduce lung inflammation and cytokine storm. The second advantage is the use of the anti-CD3 moiety to activate T cells, which makes the difference between our strategy and monoclonal, bispecific or pre-mixed neutralization antibodies. T cells are critical in eliminating virus-infected cells and tumor cells with high sensitivity. By activating T cells, S-BiTE is much more effective in eliminating virus-infected cells than antibody-mediated cytotoxicity. In the current design, we did not include an Fc portion in S-BiTE, which resulted in a short half-life in vivo. The function of S-BiTE in vivo may be enhanced by further engineering with Fc to improve its half-life. In addition, because they involve different design concepts, it is possible to develop combinational therapy including both neutralization antibodies and S-BiTE: neutralization antibodies would be focused on preventing viral spread and S-BiTE would be focused on eliminating virus-productive source cells. A similar strategy can be applied with a combination of viral-replication inhibitors and S-BiTE. Furthermore, although we used ACE2 as a targeting moiety, we believe that antibodies against spikes can also be used as targeting moieties. Binding to conserved epitopes and binding-induced T cell activation are key in engineering antibody-based BiTE against SARS-CoV-2 infection. A similar approach has demonstrated ACE2-anti-CD3 fusion protein can trigger effective CD8 T cell activation in vitro against spike-expressing cell line^56^. Our work has demonstrated it’s sufficient in activating T cells and its efficacy in controlling live SARS-CoV2 infection both in vitro and in vivo.

One limitation of our study is the using of ACE2 transgenic mice and lack of human clinical supporting data. The ACE2 expression is under the control of ubiquitous promoter, which may not reflect the physiological distribution of ACE2. An alternative approach would involve studies in human ACE2 knock-in mice. However, because we focused on spike-mediated virus neutralization and T cell activation rather than the viral life cycle, we would predict the efficacy would be similar in human ACE2 knock-in mice. Nonetheless, knowledge on the infection rate of alveolar epithelial type II cells and other ACE2^+^ cells in ACE2 knock-in mice at different infection stages will provide valuable insights from the perspective of evaluating efficacy and safety^57^.

In summary, S-BiTE can be used in a T cell-based strategy with the capability to neutralize viruses and to eliminate virus-producing cells. Further optimization of BiTE molecule stability, target moiety selection, neutralization capability, safety, and combination strategies with anti-inflammatory therapies are warranted to improve the clinical value of the S-BiTE approach in controlling SARS-CoV-2 infection.

## Methods

### Cell lines and reagents

Lenti-X 293 T cells were purchased from Clontech (Mountain View, CA, USA). The A549 cell line was obtained from the American Type Culture Collection (Manassas, VA, USA). Raji cells were provided by the Stem Cell Bank, Chinese Academy of Sciences (Shanghai, China). Human PBMCs from cord blood, NK cells, and MSCs were provided by Shanghai Longyao Biotechnology Co., Ltd. (Shanghai, China). Plasmids encoding SARS-CoV-2 spike and ACE2 were obtained from Molecular Cloud (Nanjing, China) and sub-cloned into pCDH-EF lentiviral vector plasmid (System Biosciences, Mountain View, CA, USA) with the puromycin-resistance marker.

To establish the SARS-CoV-2 spike-, SARS-CoV-2 Delta spike-, or ACE2-expressing cell lines, Lenti-X 293 T, A549, or Raji cells were infected with the SARS-CoV-2 spike-, SARS-CoV-2 Delta spike-, or ACE2-expressing lentivirus. After selection with puromycin, the pooled resistant cells were identified by flow cytometry analysis. The cell culture medium was supplemented with 10% heat-inactivated fetal bovine serum (FBS), 2 mmol/L L-glutamine, 100 units/mL penicillin, and 100 μg/mL streptomycin. Lenti-X 293 T cells, A549 cells, and their derivatives were cultured in complete DMEM. Raji cells and their derivatives were cultured in complete RPMI medium.

### Production of S-BiTE, ACE2-His, ACE2-Fc, and RBD-Fc fusion proteins

For the production of S-BiTE, ACE2-Fc, and RBD-Fc fusion proteins, DNA sequences encoding the indicated proteins were cloned into the pCDH-EF vector (System Biosciences). Plasmids containing the indicated fusion protein were transfected into Lenti-X 293 T cells, and supernatants were collected and purified using a Diamond Protein A or Ni Bestarose FF column according to the manufacturer’s protocol (Bestchrom, Shanghai, China).

### Neutralization assay with pseudotyped SARS-CoV-2

Lenti-X 293 T cells were transfected with lentivirus package component plasmids, Gap/pol (#12251, Addgene), RSV-Rev (#12253, Addgene), pCDH-EF-IRFP-luc, and pcDNA3.1(+)-2019-nCoV-spike-P2A-eGFP (#MC_0101087, Molecular Cloud). Supernatants containing lentivirus particles were collected 48 and 72 h post transfection for direct usage or concentration by ultracentrifugation. The viral titer in TU/mL was determined by flow cytometric analysis of the transduced 293-ACE2 cells.

In the virus neutralization assay, S-BiTE was serially diluted to the indicated concentrations in complete Dulbecco’s modified Eagle medium (DMEM). Pseudotyped lentiviral particles were inoculated on 293-ACE2 or A549-ACE2 monolayers in 96-well plates in the presence of 10 μg/mL of polybrene and indicated concentration of S-BiTE, and further incubated at 37 °C for 48 h. For infecting A549-ACE2 cells, a 60-min spin at 2500 rpm at 32 °C was used to improve infection efficiency. IRFP reporter activity was measured using CytoFLEX S (Beckman Coulter). The percentage of infectivity was calculated as the ratio of the IRFP readout in the presence of the fusion protein to the IRFP readout in the absence of the fusion protein. The half-maximal inhibitory concentrations (IC50) were determined using a 4-parameter logistic regression (GraphPad Prism, version 8).

### Flow cytometry

Single-cell suspensions were stained with conjugated antibodies. Samples from in vivo experiments were pre-incubated with anti-CD16/32 (anti-FcγIII/II receptor, clone 2.4G2) for 10 min before antibody staining. All fluorescently labeled monoclonal antibodies were purchased from Biolegend (San Diego, CA, USA) or eBioscience (San Diego, CA, USA). All fluorescently labeled secondary antibodies were purchased from Jackson ImmunoResearch Laboratories (West Grove, PA, USA). Samples were analyzed on a CytoFLEX S (Beckman Coulter), and data were analyzed using FlowJo software (TreeStar, Inc.).

### Enzyme-linked immunosorbent assay (ELISA) and flow cytometry analysis of S-BiTE

ELISA plates (Jet Biofil, Guangzhou, China) were coated with 2 μg/mL of RBD-Fc at 4 °C overnight. Plates were washed three times with phosphate buffered saline (PBS) containing 0.05% Tween-20 and blocked with 2% FBS in PBS at room temperature for 1 h. Diluted S-BiTE-containing samples were added, and plates were incubated for 1 h at room temperature. Then, plates were washed three times and incubated for 1 h at room temperature with alkaline phosphatase (AP)-conjugated goat anti-mouse Fab secondary antibody (Jackson ImmunoResearch Laboratories) diluted to 1:2000 in blocking buffer. AP activity was measured at 405 nm using a SpectraMax 190 microplate reader (Molecular Devices, San Jose, CA) with p-nitrophenyl phosphate (Guangzhou Howei Pharmaceutical Technology Co. Ltd., Guangzhou, China) as substrate. The half-maximum effective concentration (EC50) binding values were calculated using GraphPad Prism version 8.

293-CD3 and human primary T cells were incubated with the indicated S-BiTE-containing samples at 4 °C for 30 min, washed three times with 2% FBS in PBS, incubated with 2 μg/mL of RBD-hFc at 4 °C for 30 min, washed three times, and incubated with 1:200 diluted Alexa Fluor 647 conjugated goat anti-human IgG Fc antibodies (Jackson ImmunoResearch Laboratories). The cells were then subjected to flow cytometric analysis.

293-spike or 293-Delta-spike cells were incubated with the indicated S-BiTE-containing samples at 4 °C for 30 min, washed three times with 2% FBS in PBS, and incubated with 1:200 diluted Alexa Fluor 647 conjugated goat anti-mouse Fab antibodies (Jackson ImmunoResearch Laboratories). The cells were then subjected to flow cytometric analysis.

### Lentivirus production

Lentivirus was produced by transient transfection of Lenti-X 293 T cells with a four-plasmid system. Supernatants containing the lentiviral particles were collected at 48 and 72 h post-transfection and used to establish stable cell lines.

### In vitro T cell activation and killing assay

Human T cells were stimulated with anti-CD3 (0.25 μg/mL; Bio X Cell, Lebanon, NH, USA) and anti-CD28 (1 μg/mL; Bio X Cell) for 2 days, rested for 3 days, and used for the in vitro activation and killing assay in complete RPMI 1640 supplemented with IL-2 (50 IU/mL) (Beijing Four Rings Bio-Pharmaceutical Co., Beijing, China) and IL-21 (4 ng/mL; Biolegend). Approximately 1 × 10^5^ T cells were co-cultured with 2.5 × 10^4^ Raji or Raji-spike cells; 3.75 × 10^4^ 293, 293-spike, or 293-Delta spike cells; or 1.25 × 10^4^ A549, A549-spike, or A549-Delta spike cells in the presence of various concentrations of S-BiTE. After 1 day, levels of TNF and IFN-γ in the supernatant were analyzed by a cytometric bead array (CBA) assay according to the manufacturer’s protocol (BD Biosciences, San Jose, CA). After 2 days, the killing assay was conducted using flow cytometry. Anti-CD45 antibody was used to distinguish T cells from 293 or A549 cells. Anti-CD3 and anti-CD19 antibodies were used to distinguish T cells from Raji-derived cells.

A549 and A549-spike cells were labeled with CFSE (MedChemExpress, Shanghai, China) and CellTrace^TM^ violet (Life Technologies Corporation, Eugene, OR, USA), respectively, according to the manufacturer’s protocol. Then, 1.5 × 10^4^ fluorescent dye-labeled A549 and A549-spike cells were plated on a 96-well plate. After 6 h, 1 × 10^5^ T cells and various concentrations of S-BiTE were added to the culture. Killing efficiency was determined using the Operetta CLS (PerkinElmer, Waltham, MA, USA).

### Pseudotyped virus release assay

Lenti-X 293 T cells were transfected with Gag/Pol (#12251, Addgene), RSV-Rev (#12253, Addgene), pcDNA3.1(+)-2019-nCoV-spike-P2A-eGFP (#MC_0101087, MolecularCloud), and pCDH-EF-GFP-luc in 24-well plates. After 24 h, 1 × 10^5^ T cells were added to the culture with or without S-BiTE stimulation. Supernatants were collected at 48 h post-transfection. The viral titer was measured by infecting 293-ACE2 cells. Levels of TNF and IFN-γ in the supernatant were analyzed by the CBA assay. The remaining virus-releasing cells were imaged by REVOLVE fluorescent microscopy (ECHO, San Diego, CA, USA) and analyzed by flow cytometry.

### Live SARS-CoV-2 infection assay in vitro and in vivo

The inhibition assay for live SARS-CoV-2 was performed in a biosafety level 3 (BSL3) facility at Fudan University. A549-ACE2 cells were seeded in 24-well plates and infected with live SARS-CoV-2 (GenBank: MT121215.1) at a multiplicity of infection (MOI) of 0.05. After 2 h, the free viruses were removed by washing with PBS. Human primary T cells were added to the culture in the presence of 1 or10 μg/mL of S-BiTE. At 24 h and 48 h post infection, cells were collected and mRNA was isolated. Reverse-transcription quantitative polymerase chain reaction (RT-qPCR) was used to test the SARS-CoV-2 mRNA viral titer using the One-Step PrimeScript RT-PCR Kit (Takara, Shiga, Japan) with the following primers:

SARS-CoV-2-N-F: GGGGAACTTCTCCTGCTAGAAT;

SARS-CoV-2-N-R: CAGACATTTTGCTCTCAAGCTG;

SARS-CoV-2-N-probe: 5’-FAM- TTGCTGCTGCTTGACAGATT-TAMRA-3’.

hACE2- hCD3ε transgenic mice were intranasally administrated with twenty-five μg of S-BiTE, equal mole of ACE2-His, or equal volume of PBS. One hour later, mice were intranasally infected with 10,000 PFU of SARS-CoV2 Delta-variant. Twenty-five μg of S-BiTE, equal mole of ACE2-His, or equal volume of PBS were administrated 24, and 48 h post-infection. Three days later, lungs and intestines of three groups collected for RNA isolation and RT-PCR.

### Mice

Six-eight weeks old female C57BL/6 J mice were purchased from Beijing Vital River Laboratory Animal Technology Co., Ltd. (Beijing, China). Eight-ten weeks old hACE2-hCD3ε transgenic mice, Six-eight weeks old female NOD-*Prkdc*^scid^*IL2rγ*^tm1^ (NSG) mice were purchased from the Shanghai Model Organisms Center, Inc. (Shanghai, China). All mice were maintained under specific pathogen-free conditions. Animal care and use were in accordance with institutional and NIH protocols and guidelines, and all studies were approved by the Animal Care and Use Committee of Shanghai Jiao Tong University and the Institutional Laboratory Animal Care of Fudan University (20220609-001).

### In vivo T cell killing assay

Raji and Raji-spike cells were labeled with 5 or 50 μM of CFSE, respectively, according to the manufacturer’s protocol (MedChemExpress). Approximately 2 × 10^6^ CFSE-labeled Raji and Raji-spike cells were mixed with 5 × 10^6^ T cells and injected intraperitoneally into NSG mice. The mice were treated with PBS or S-BiTE, and cells in the peritoneal cavity were collected and analyzed by flow cytometry 6 h after treatment.

### Statistics and reproducibility

The number of independent biological replicates (n) of each experiment was noted in the figure legends. All attempts at replication were successful. All statistical analyses were performed using GraphPad Prism 8. Error bars represent standard deviation (SD) or standard error of the mean (SEM). Statistical analyses were performed using the Student’s t-test and one-way or two-way analysis of variance (ANOVA) with Dunnett multiple comparisons correction.

### Reporting summary

Further information on research design is available in the Nature Portfolio Reporting Summary linked to this article.

## Supplementary information

Peer Review File

Supplementary information

Description of Additional Supplementary Files

Supplementary Data 1

Reporting Summary

## Data Availability

The main data supporting the results in this study are available within the paper and its Supplementary Information. Source data for all figures can be found in Supplementary Data 1. The raw and analysed datasets generated during the study are too large to be publicly shared, yet they are available for research purposes from the corresponding authors on reasonable request.

## References

[1] Zhou P (2020). A pneumonia outbreak associated with a new coronavirus of probable bat origin. Nature.

[2] Guan WJ (2020). Clinical characteristics of coronavirus disease 2019 in China. N. Engl. J. Med..

[3] Coronaviridae Study Group of the International Committee on Taxonomy of, V. (2020). The species Severe acute respiratory syndrome-related coronavirus: classifying 2019-nCoV and naming it SARS-CoV-2. Nat. Microbiol..

[4] Jara, A. et al. Effectiveness of an inactivated SARS-CoV-2 vaccine in Chile. *N. Engl. J. Med*. 10.1056/NEJMoa2107715 (2021). 10.1056/NEJMoa2107715 PMC8279092 34233097

[5] Polack FP (2020). Safety and efficacy of the BNT162b2 mRNA Covid-19 vaccine. N. Engl. J. Med..

[6] Sadoff J (2021). Safety and efficacy of single-dose Ad26.COV2.S vaccine against Covid-19. N. Engl. J. Med..

[7] Davies, N. G. et al. Estimated transmissibility and impact of SARS-CoV-2 lineage B.1.1.7 in England. *Science* **372**, eabg3055 (2021). 10.1126/science.abg3055 PMC8128288 33658326

[8] Wang Z (2021). mRNA vaccine-elicited antibodies to SARS-CoV-2 and circulating variants. Nature.

[9] Xiang, R. et al. Recent advances in developing small-molecule inhibitors against SARS-CoV-2. *Acta Pharm Sin B*10.1016/j.apsb.2021.06.016 (2021). 10.1016/j.apsb.2021.06.016 PMC8260826 34249607

[10] Nepali K, Sharma R, Sharma S, Thakur A, Liou JP (2022). Beyond the vaccines: a glance at the small molecule and peptide-based anti-COVID19 arsenal. J. Biomed. Sci..

[11] Dryden-Peterson S (2023). Nirmatrelvir plus ritonavir for early COVID-19 in a large U.S. health system: a population-based cohort study. Ann. Intern Med..

[12] Hammond J (2022). Oral nirmatrelvir for high-risk, nonhospitalized adults with Covid-19. N. Engl. J. Med..

[13] Ganatra, S. et al. Oral nirmatrelvir and ritonavir in non-hospitalized vaccinated patients with Covid-19. *Clin. Infect. Dis.*10.1093/cid/ciac673 (2022).

[14] Chen, X. et al. Human monoclonal antibodies block the binding of SARS-CoV-2 spike protein to angiotensin converting enzyme 2 receptor. *Cell. Mol. Immunol.*10.1038/s41423-020-0426-7 (2020). 10.1038/s41423-020-0426-7 PMC7167496 32313207

[15] Wang C (2020). A human monoclonal antibody blocking SARS-CoV-2 infection. Nat. Commun..

[16] Shi R (2020). A human neutralizing antibody targets the receptor-binding site of SARS-CoV-2. Nature.

[17] Cao Y (2020). Potent neutralizing antibodies against SARS-CoV-2 identified by high-throughput single-cell sequencing of convalescent patients’ B cells. Cell.

[18] Chi X (2020). A neutralizing human antibody binds to the N-terminal domain of the Spike protein of SARS-CoV-2. Science.

[19] Weinreich DM (2021). REGN-COV2, a neutralizing antibody cocktail, in outpatients with Covid-19. N. Engl. J. Med..

[20] Lei C (2020). Neutralization of SARS-CoV-2 spike pseudotyped virus by recombinant ACE2-Ig. Nat. Commun..

[21] Glasgow A (2020). Engineered ACE2 receptor traps potently neutralize SARS-CoV-2. Proc. Natl Acad. Sci. USA.

[22] Chan KK (2020). Engineering human ACE2 to optimize binding to the spike protein of SARS coronavirus 2. Science.

[23] Monteil, V. et al. Inhibition of SARS-CoV-2 infections in engineered human tissues using clinical-grade soluble human ACE2. *Cell*10.1016/j.cell.2020.04.004 (2020). 10.1016/j.cell.2020.04.004 PMC7181998 32333836

[24] Cho H (2021). Bispecific antibodies targeting distinct regions of the spike protein potently neutralize SARS-CoV-2 variants of concern. Sci. Transl. Med..

[25] De Gasparo R (2021). Bispecific IgG neutralizes SARS-CoV-2 variants and prevents escape in mice. Nature.

[26] Li Z (2022). An engineered bispecific human monoclonal antibody against SARS-CoV-2. Nat. Immunol..

[27] Hanke L (2022). A bispecific monomeric nanobody induces spike trimer dimers and neutralizes SARS-CoV-2 in vivo. Nat. Commun..

[28] Qian K, Hu S (2020). Ig-like ACE2 protein therapeutics: A revival in development during the COVID-19 pandemic. MAbs.

[29] Miao X (2020). A novel biparatopic hybrid antibody-ACE2 fusion that blocks SARS-CoV-2 infection: implications for therapy. MAbs.

[30] Edara VV (2021). Infection and Vaccine-Induced Neutralizing-Antibody Responses to the SARS-CoV-2 B.1.617 Variants. N. Engl. J. Med..

[31] Lopez Bernal J (2021). Effectiveness of Covid-19 Vaccines against the B.1.617.2 (Delta) Variant. N. Engl. J. Med..

[32] Garcia-Beltran WF (2021). Multiple SARS-CoV-2 variants escape neutralization by vaccine-induced humoral immunity. Cell.

[33] Tada T (2021). Convalescent-phase sera and vaccine-elicited antibodies largely maintain neutralizing titer against global SARS-CoV-2 variant spikes. mBio.

[34] Wang P (2021). Antibody resistance of SARS-CoV-2 variants B.1.351 and B.1.1.7. Nature.

[35] Wu K (2021). Serum neutralizing activity elicited by mRNA-1273 vaccine. N. Engl. J. Med..

[36] Xie X (2021). Neutralization of SARS-CoV-2 spike 69/70 deletion, E484K and N501Y variants by BNT162b2 vaccine-elicited sera. Nat. Med..

[37] Liu J (2021). BNT162b2-elicited neutralization of B.1.617 and other SARS-CoV-2 variants. Nature.

[38] Planas D (2021). Reduced sensitivity of SARS-CoV-2 variant Delta to antibody neutralization. Nature.

[39] Leen AM, Rooney CM, Foster AE (2007). Improving T cell therapy for cancer. Annu Rev. Immunol..

[40] Barry M, Bleackley RC (2002). Cytotoxic T lymphocytes: all roads lead to death. Nat. Rev. Immunol..

[41] Baeuerle PA, Reinhardt C (2009). Bispecific T-cell engaging antibodies for cancer therapy. Cancer Res..

[42] Hoffmann M (2020). SARS-CoV-2 cell entry depends on ACE2 and TMPRSS2 and is blocked by a clinically proven protease inhibitor. Cell.

[43] Yan R (2020). Structural basis for the recognition of SARS-CoV-2 by full-length human ACE2. Science.

[44] Staflin, K. et al. Target arm affinities determine preclinical efficacy and safety of anti-HER2/CD3 bispecific antibody. *JCI Insight* **5**10.1172/jci.insight.133757 (2020). 10.1172/jci.insight.133757 PMC7205277 32271166

[45] Bortoletto N, Scotet E, Myamoto Y, D’Oro U, Lanzavecchia A (2002). Optimizing anti-CD3 affinity for effective T cell targeting against tumor cells. Eur. J. Immunol..

[46] Wu, Y. et al. A noncompeting pair of human neutralizing antibodies block COVID-19 virus binding to its receptor ACE2. *Science*10.1126/science.abc2241 (2020). 10.1126/science.abc2241 PMC7223722 32404477

[47] Pinto, D. et al. Cross-neutralization of SARS-CoV-2 by a human monoclonal SARS-CoV antibody. *Nature*10.1038/s41586-020-2349-y (2020). 10.1038/s41586-020-2349-y 32422645

[48] Salazar G, Zhang N, Fu TM, An Z (2017). Antibody therapies for the prevention and treatment of viral infections. NPJ Vaccines.

[49] Cao, Y. et al. Potent neutralizing antibodies against SARS-CoV-2 identified by high-throughput single-cell sequencing of convalescent patients’ B cells. *Cell*10.1016/j.cell.2020.05.025 (2020). 10.1016/j.cell.2020.05.025 PMC7231725 32425270

[50] Nimmerjahn F, Ravetch JV (2006). Fcgamma receptors: old friends and new family members. Immunity.

[51] Frenette PS, Pinho S, Lucas D, Scheiermann C (2013). Mesenchymal stem cell: keystone of the hematopoietic stem cell niche and a stepping-stone for regenerative medicine. Annu Rev. Immunol..

[52] Public Health England. Variants distribution of cases. https://www.gov.uk/government/publications/covid-19-variants-genomically-confirmed-case-numbers/variants-distribution-of-case-data-11-june-2021 (2021).

[53] Baum A (2020). Antibody cocktail to SARS-CoV-2 spike protein prevents rapid mutational escape seen with individual antibodies. Science.

[54] Sun Y (2021). Structure-based development of three- and four-antibody cocktails against SARS-CoV-2 via multiple mechanisms. Cell Res..

[55] Cho, H. et al. Ultrapotent bispecific antibodies neutralize emerging SARS-CoV-2 variants. *bioRxiv*10.1101/2021.04.01.437942 (2021).

[56] Dogan M (2022). Targeting SARS-CoV-2 infection through CAR-T-like bispecific T cell engagers incorporating ACE2. Clin. Transl. Immunol..

[57] Zou, X. et al. Single-cell RNA-seq data analysis on the receptor ACE2 expression reveals the potential risk of different human organs vulnerable to 2019-nCoV infection. *Front. Med.*10.1007/s11684-020-0754-0 (2020). 10.1007/s11684-020-0754-0 PMC7088738 32170560

