# Supplementary material for: A spike-targeting bispecific T cell engager strategy provides dual layer protection against SARS-CoV-2 infection in vivo
Source: Commun Biol. 2023 Jun 1;6:592. doi: 10.1038/s42003-023-04955-3 (PMC10234585; doi:10.1038/s42003-023-04955-3)
Supplement: Supplementary file 2 — Supplementary information [file 42003_2023_4955_MOESM2_ESM.pdf]

Supplementary information

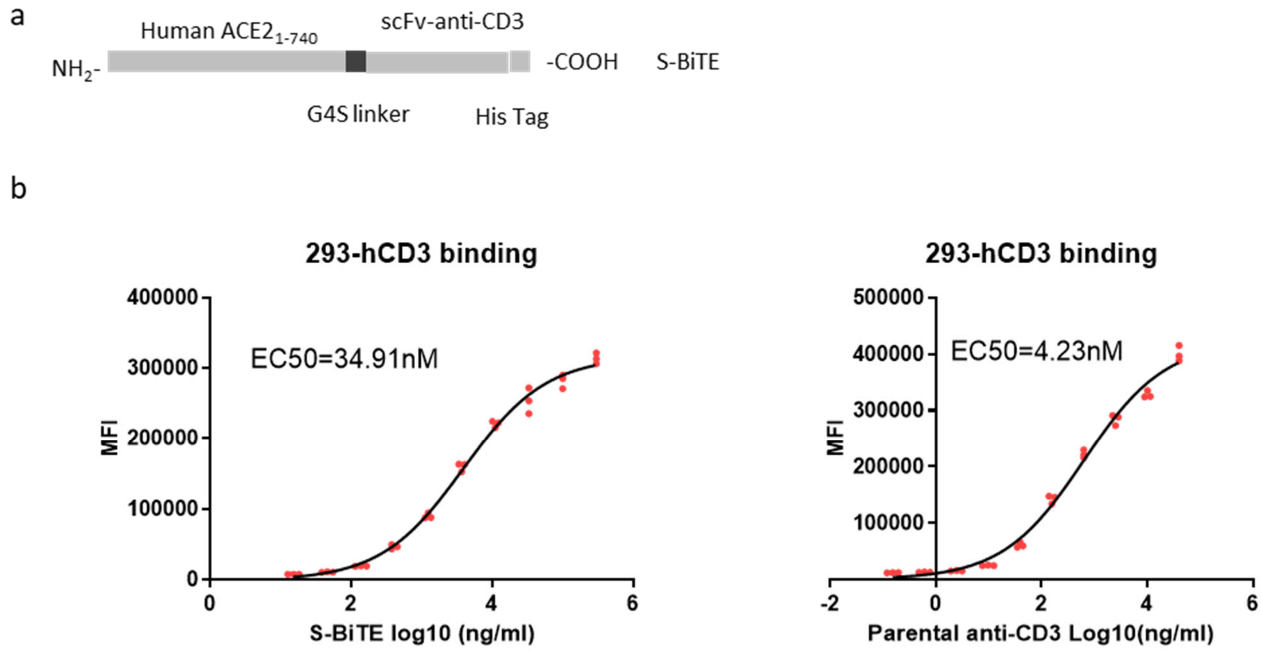

Supplementary Fig. 1. Characterization of S-BiTE. a, a schematic diagram of the ACE2-scFv-anti-CD3 (S-BiTE) fusion protein. b, MFI of binding of S-BiTE or parental anti-CD3 to 293-CD3 cells (n=3/group).

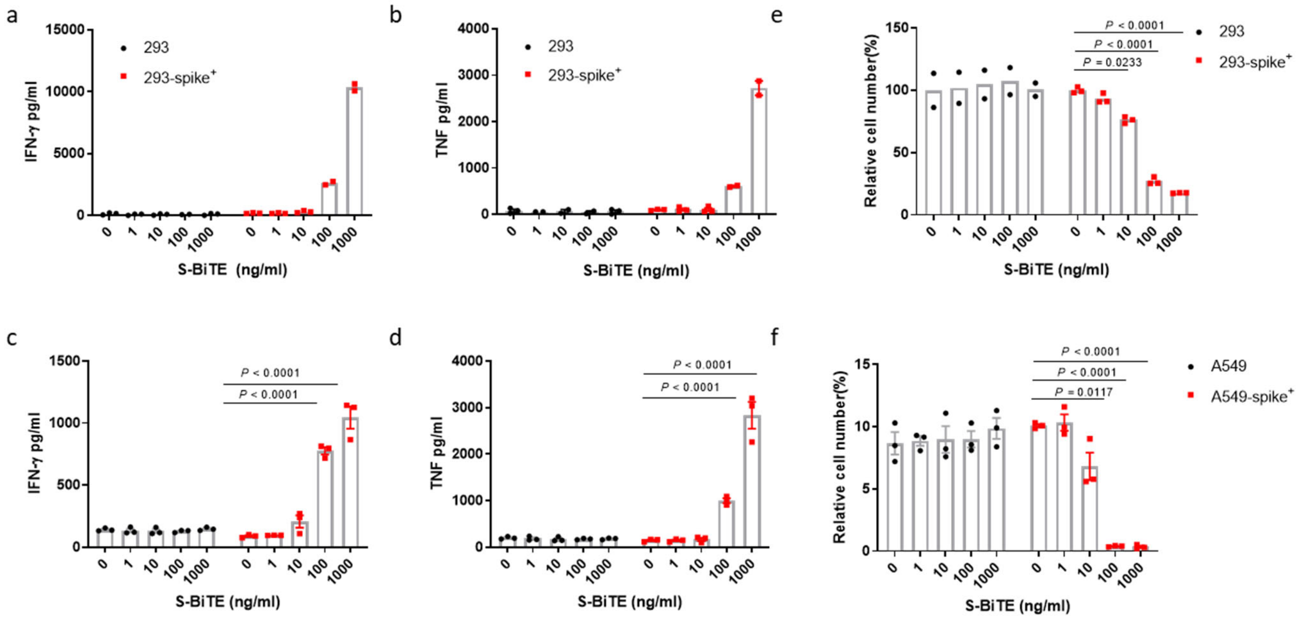

Supplementary Fig. 2. S-BiTE induced potent cytotoxicity of T cells. 293, 293-spike, A549, or A549-spike cells were co-cultured with human primary T cell in the presence of indicated concentration of S-BiTE. Twenty-four hours later, IFN-γ and TNF in supernatant were analyzed by CBA assay (a-d) (n=2-3/group, Error bars represent SEM). Forty-eight hours later, cytotoxicity was determined by measuring $CD45^-$ cells by flow cytometry (e-f) (n=2-3/group, Error bars represent SEM).

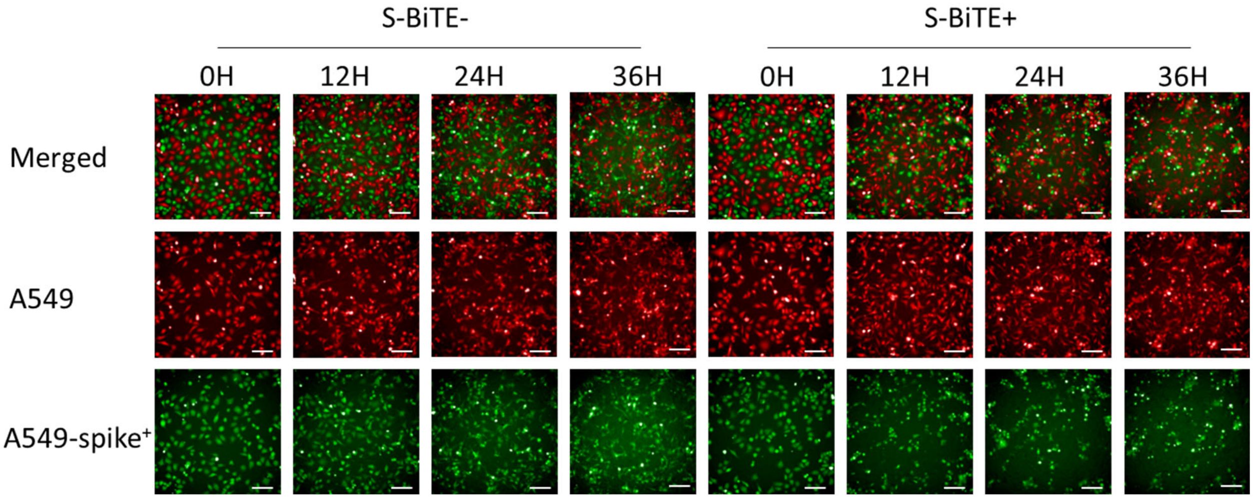

Supplementary Fig. 3. S-BiTE specifically killed spike^{+} cell in the presence of T cells. CellTrace Violet-labeled A549 and CFSE-labeled A549-spike^{+} cells were co-cultured with human primary T cell in the absence or presence of 1μg/ml of S-BiTE. The cytotoxicity was determined by Operetta CLS at indicated time points. Scale bar: 120 μm.

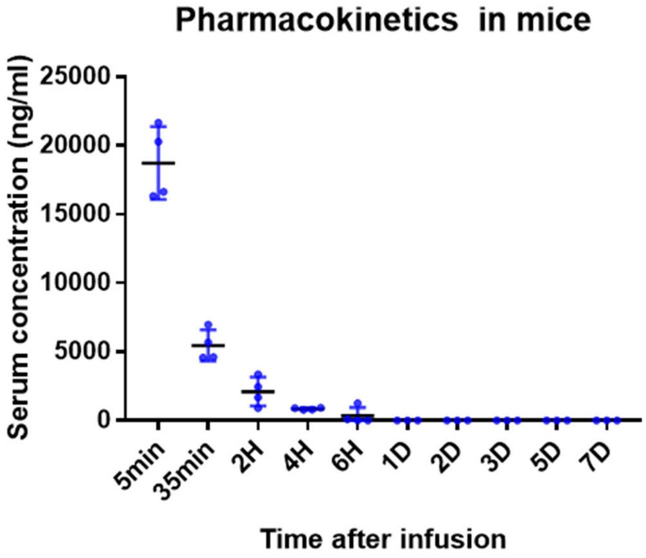

Supplementary Fig. 4. In vivo persistence analysis of S-BiTE. C57BL/6 mice received a single injection of 25 μg S-BiTE and serum concentrations were determined by ELISA at indicated time points (n=5/group, Error bars represent SEM).

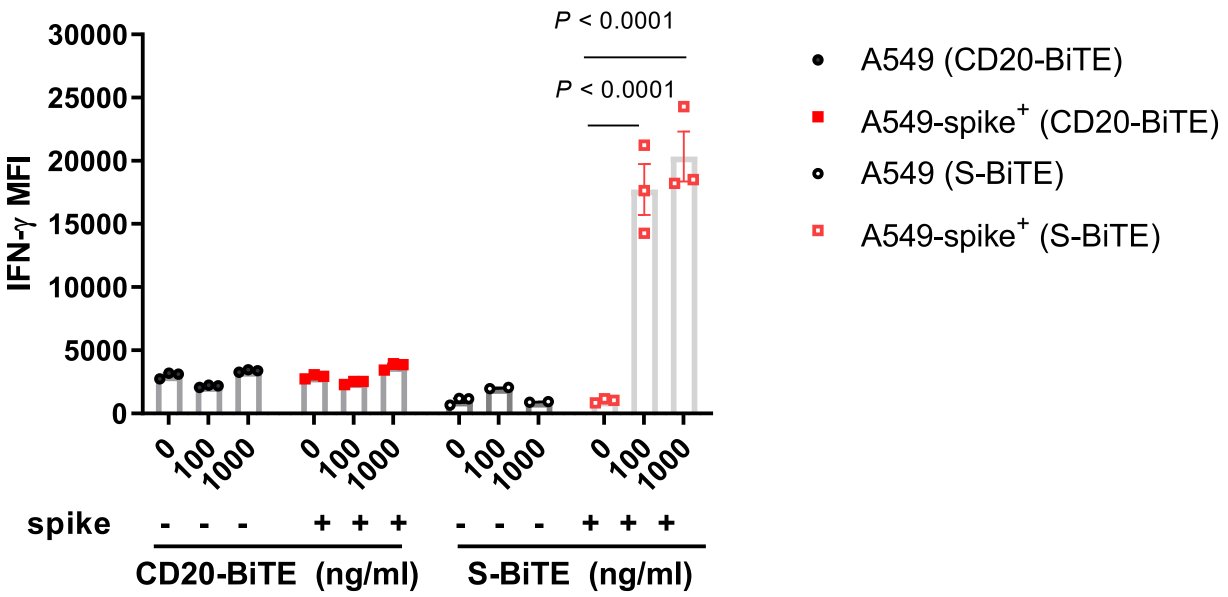

Supplementary Fig. 5. S-BiTE induced target-dependent T cell activation in the presence of spike. A549, A549-spike were co-cultured with human primary T cells in the presence of the indicated concentration of CD20-targeting CD20-BiTE or spike-targeting S-BiTE. After 24 h, levels of IFN-γ in the cell supernatant were analyzed by the CBA assay (n=2-3/group, Error bars represent SEM).

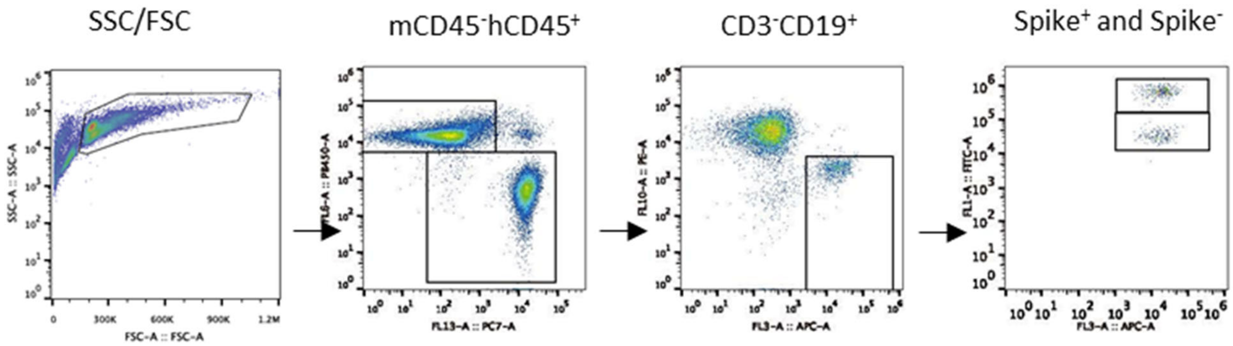

Supplementary Fig. 6. Gating Strategy of flow cytometry analysis.

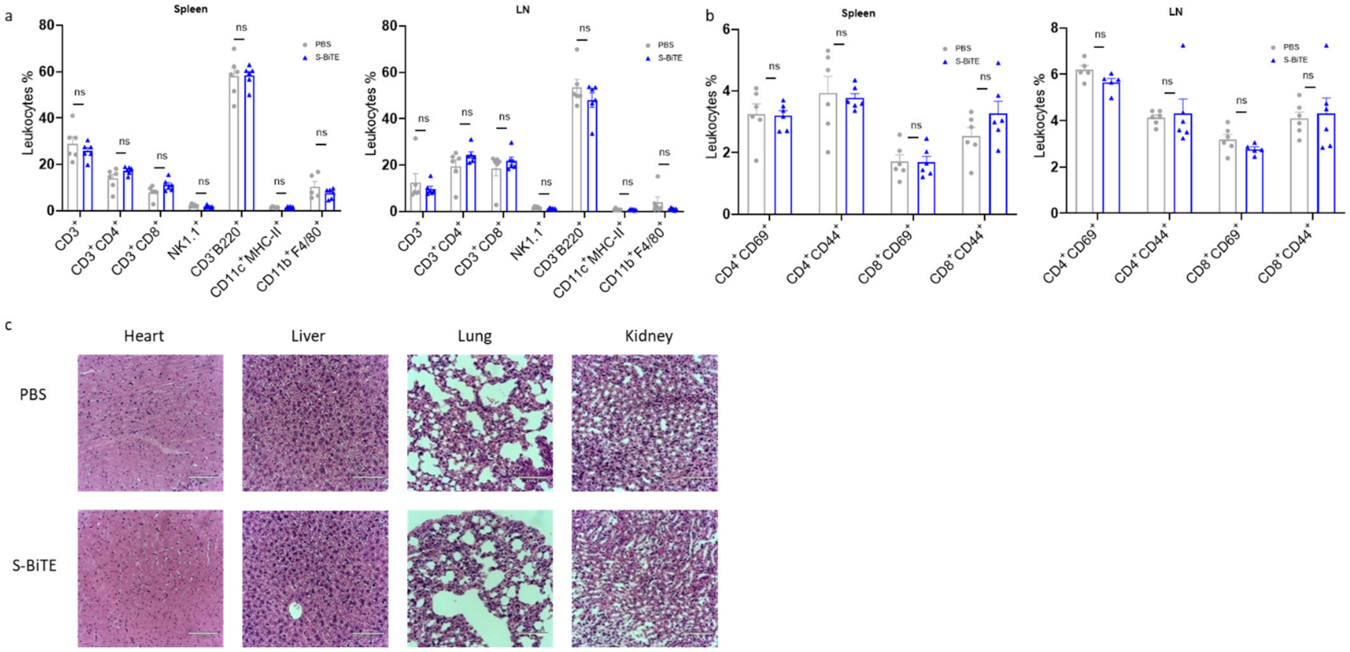

Supplementary Fig. 7. In vivo safety profile of S-BiTE. a. hACE2-hCD3ε mice (n=6) were administrated with 150 μg of S-BiTE or PBS on day 0 and day 3. Six days later, single cell suspension was prepared and analyzed by flow cytometry. $CD3^+$, $CD3^+CD4^+$, $CD3^+CD8^+$, $NK1.1^+$, $CD3^-B220^+$ cell percentage was analyzed. Error bars represent SEM. b. The expression of CD44 and CD69 in $CD3^+CD4^+$ and $CD3^+CD8^+$ was analyzed by flow cytometry (n=6). Error bars represent SEM. c. Heart, liver, lung and kidney was collected for HE staining. Scale bar: 100μm.
