# Supplementary material for: A spike-targeting bispecific T cell engager strategy provides dual layer protection against SARS-CoV-2 infection in vivo
Source: Commun Biol. 2023 Jun 1;6:592. doi: 10.1038/s42003-023-04955-3 (PMC10234585; doi:10.1038/s42003-023-04955-3)
Supplement: Supplementary file 5 — Reporting Summary [file 42003_2023_4955_MOESM5_ESM.pdf]

nature research

Corresponding author(s): Xuanming Yang

Last updated by author(s): Apr 20, 2023

Reporting Summary

Nature Research wishes to improve the reproducibility of the work that we publish. This form provides structure for consistency and transparency in reporting. For further information on Nature Research policies, see our Editorial Policies and the Editorial Policy Checklist.

Statistics

For all statistical analyses, confirm that the following items are present in the figure legend, table legend, main text, or Methods section.

n/a	Confirmed	
☐	☒	The exact sample size (n) for each experimental group/condition, given as a discrete number and unit of measurement
☐	☒	A statement on whether measurements were taken from distinct samples or whether the same sample was measured repeatedly
☐	☒	The statistical test(s) used AND whether they are one- or two-sided<br>*Only common tests should be described solely by name; describe more complex techniques in the Methods section.*
☒	☐	A description of all covariates tested
☐	☒	A description of any assumptions or corrections, such as tests of normality and adjustment for multiple comparisons
☒	☐	A full description of the statistical parameters including central tendency (e.g. means) or other basic estimates (e.g. regression coefficient) AND variation (e.g. standard deviation) or associated estimates of uncertainty (e.g. confidence intervals)
☐	☒	For null hypothesis testing, the test statistic (e.g. F, t, r) with confidence intervals, effect sizes, degrees of freedom and P value noted<br>*Give P values as exact values whenever suitable.*
☒	☐	For Bayesian analysis, information on the choice of priors and Markov chain Monte Carlo settings
☒	☐	For hierarchical and complex designs, identification of the appropriate level for tests and full reporting of outcomes
☒	☐	Estimates of effect sizes (e.g. Cohen's d, Pearson's r), indicating how they were calculated

Our web collection on statistics for biologists contains articles on many of the points above.

Software and code

Policy information about availability of computer code

Data collection	No sequencing data were generated in this study.
Data analysis	No sequencing data were analyzed in this study.

For manuscripts utilizing custom algorithms or software that are central to the research but not yet described in published literature, software must be made available to editors and reviewers. We strongly encourage code deposition in a community repository (e.g. GitHub). See the Nature Research guidelines for submitting code & software for further information.

Data

Policy information about availability of data

All manuscripts must include a data availability statement. This statement should provide the following information, where applicable:

- Accession codes, unique identifiers, or web links for publicly available datasets
- A list of figures that have associated raw data
- A description of any restrictions on data availability

The authors declare that all the data supporting the findings of this study are available from the authors on reasonable request.

Field-specific reporting

Please select the one below that is the best fit for your research. If you are not sure, read the appropriate sections before making your selection.

☒ Life sciences ☐ Behavioural & social sciences ☐ Ecological, evolutionary & environmental sciences

For a reference copy of the document with all sections, see nature.com/documents/nr-reporting-summary-flat.pdf

Life sciences study design

All studies must disclose on these points even when the disclosure is negative.

Sample size	We did not predetermine sample size. All experiments were performed multiple times. If results were reproducible, the conclusions were consider valid.
Data exclusions	No data were excluded.
Replication	All attempts at replication were successful.
Randomization	Samples were not randomized.
Blinding	The investigators were not blinded to group allocation during data collection and analysis.

Reporting for specific materials, systems and methods

We require information from authors about some types of materials, experimental systems and methods used in many studies. Here, indicate whether each material, system or method listed is relevant to your study. If you are not sure if a list item applies to your research, read the appropriate section before selecting a response.

Materials & experimental systems

n/a	Involved in the study	
☐	☒	Antibodies
☐	☒	Eukaryotic cell lines
☒	☐	Palaeontology and archaeology
☐	☒	Animals and other organisms
☒	☐	Human research participants
☒	☐	Clinical data
☒	☐	Dual use research of concern

Methods

n/a	Involved in the study	
☒	☐	ChIP-seq
☐	☒	Flow cytometry
☒	☐	MRI-based neuroimaging

Antibodies

Antibodies used	Alexa Fluor 647 conjugated goat anti-human IgG Fc antibodies (Jackson ImmunoResearch Laboratories); FITC conjugated anti-human CD3 (clone OKT3,(Biolegend); Pacific blue conjugated anti-human CD45 (Biolegend).
Validation	These antibodies are validated by the provider and have passed the routine QC test by the manufacturer.

Eukaryotic cell lines

Policy information about cell lines

Cell line source(s)	Lenti-X 293T cells were purchased from Clontech (Mountain View, CA, USA). The A549 cell line was obtained from the American Type Culture Collection (Manassas, VA, USA). Raji cells were provided by the Stem Cell Bank, Chinese Academy of Sciences (Shanghai, China).
Authentication	None of the cell lines used were authenticated.
Mycoplasma contamination	All cell lines are tested negative for mycoplasma.
Commonly misidentified lines (See ICLAC register)	No commonly misidentified lines were used in this study.

Animals and other organisms

Policy information about studies involving animals; ARRIVE guidelines recommended for reporting animal research

Laboratory animals	NOD-PrkdcscidIL2rytm1 mice were purchased from Shanghai Model Organisms Center, Inc. C57BL/6 mice were obtained from Beijing Vital River Laboratory Animal Technology Co., Ltd (Beijing, China).
Wild animals	No wild animals were used in this study.
Field-collected samples	No field-collected samples were used in this study.
Ethics oversight	All mice were maintained under SPF conditions. Animal care and use were in accordance with institutional and NIH protocols and guidelines, and all studies were approved by the Animal Care and Use Committee of Shanghai Jiao Tong University.

Note that full information on the approval of the study protocol must also be provided in the manuscript.

Flow Cytometry

Plots

Confirm that:

☒ The axis labels state the marker and fluorochrome used (e.g. CD4-FITC).

☒ The axis scales are clearly visible. Include numbers along axes only for bottom left plot of group (a 'group' is an analysis of identical markers).

☒ All plots are contour plots with outliers or pseudocolor plots.

☒ A numerical value for number of cells or percentage (with statistics) is provided.

Methodology

Sample preparation	For cell-surface staining, cells were incubated for 10 min in staining buffer (1× phosphate-buffered saline [PBS] with 1% FBS) with antibodies recognizing CD16 and CD32 (anti-FcγRII and Fc�RIII, clone 2.4 G2), or Human TruStain FcX (BioLegend) to block nonspecific Fc-mediated binding. The blocked samples were subsequently stained with conjugated antibodies. The cells were analyzed using a CytoFLEX S flow cytometer (Beckman Coulter), and the data were analyzed with FlowJo software (Becton Dickinson).
Instrument	Cytoflex S (Beckman Coulter)
Software	Flowjo
Cell population abundance	Cell population abundance was not analyzed in this study.
Gating strategy	Cells were firstly gated based on FSC/SSC. Then hCD45+mCD45- negative populations were gated for hCD19 and hCD3 staining analysis. Then hCD3-hCD19+ populations were gated for CFSE analysis.

☒ Tick this box to confirm that a figure exemplifying the gating strategy is provided in the Supplementary Information.
