# Supplementary material for: A spike-targeting bispecific T cell engager strategy provides dual layer protection against SARS-CoV-2 infection in vivo
Source: Commun Biol. 2023 Jun 1;6:592. doi: 10.1038/s42003-023-04955-3 (PMC10234585; doi:10.1038/s42003-023-04955-3)
Supplement: Supplementary file 1 — Peer Review File [file 42003_2023_4955_MOESM1_ESM.pdf]

nature portfolio

Peer Review File

A spike-targeting bispecific T cell engager strategy provides dual layer protection against SARS-CoV-2 infection in vivo

Open Access This file is licensed under a Creative Commons Attribution 4.0 International License, which permits use, sharing, adaptation, distribution and reproduction in any medium or format, as long as you give appropriate credit to the original author(s) and the source, provide a link to the Creative Commons license, and indicate if changes were made. In the cases where the authors are anonymous, such as is the case for the reports of anonymous peer reviewers, author attribution should be to 'Anonymous Referee' followed by a clear attribution to the source work. The images or other third party material in this file are included in the article's Creative Commons license, unless indicated otherwise in a credit line to the material. If material is not included in the article's Creative Commons license and your intended use is not permitted by statutory regulation or exceeds the permitted use, you will need to obtain permission directly from the copyright holder. To view a copy of this license, visit http://creativecommons.org/licenses/by/4.0/.

REVIEWERS' COMMENTS:

Reviewer #1 (Remarks to the Author):

I am satisfied with the answers and editing provided by the Authors.

Reviewer #2 (Remarks to the Author):

The authors have responded well to the critiques and have materially improved the paper. It is now acceptable.

Reviewer #3 (Remarks to the Author):

To whom it may concern:

the authors addressed all the points I had. Even though the Covid pandemic is over, it is a very nice piece of work illustrating the potential of BiTEs for treating viral diseases.
